# Analysis of Carcinogenic Involvement of MicroRNA Pattern in Peripheral Non-Cancerous Tissues and Chronic Viral Liver Injury

**DOI:** 10.3390/ijms25147858

**Published:** 2024-07-18

**Authors:** Tomohiro Umezu, Tomoya Mori, Hidenori Toyoda, Kohsuke Kanekura, Akihiro Tamori, Takahiro Ochiya, Masahiko Kuroda, Tatsuya Akutsu, Yoshiki Murakami

**Affiliations:** 1 Department of Molecular Pathology, Tokyo Medical University, 6-1-1 Shinjuku, Shinjuku-ku, Tokyo 160-8402, Japan; t_umezu@tokyo-med.ac.jp (T.U.); kanekura@tokyo-med.ac.jp (K.K.); kuroda@tokyo-med.ac.jp (M.K.); 2 Bioinformatics Center, Institute for Chemical Research, Kyoto University, Gokasho, Uji, Kyoto 611-0011, Japan; tmori@kuicr.kyoto-u.ac.jp (T.M.); takutsu@kuicr.kyoto-u.ac.jp (T.A.); 3 Department of Gastroenterology, Ogaki Municipal Hospital, 4-86 Minaminokawa-cho, Ogaki, Gifu 503-8502, Japan; hmtoyoda@spice.ocn.ne.jp; 4 Department of Hepatology, Osaka Metropolitan University Graduate School of Medicine, 1-4-3 Asahimachi, Abeno-ku, Osaka 545-8585, Japan; tamori-a@omu.ac.jp; 5 Institution of Medical Science, Tokyo Medical University, 6-7-1 Nishishinjuku, Shinjuku-ku, Tokyo 160-0023, Japan; tochiya@tokyo-med.ac.jp; 6 Department of Dentistry, Asahi University, 3-23 Hashimoto-cho, Gifu 500-8523, Japan

**Keywords:** miRNA, hepatocellular carcinoma, HCV, surrounding non-tumor tissues, chronic hepatitis

## Abstract

Risk factors for hepatocarcinogenesis include chronic inflammation due to viral infection, liver fibrosis, and aging. In this study, we separated carcinogenic and non-carcinogenic cases due to hepatitis C virus (HCV) infection, aiming to comprehensively analyze miRNA expression in liver tissues by age, and identify factors that contribute to carcinogenesis. Total RNA was extracted from 360 chronic hepatitis C (CH), 43 HCV infected hepatocellular carcinoma (HCC), and surrounding non-tumor (SNT) tissues. MicroRNA (miRNA) expression patterns were analyzed using microarray. Using machine learning, we extracted characteristic miRNA expression patterns for each disease and age. There were no age-dependent changes in miRNA expression in the disease-specific comparisons; however, miRNA expression differed among the age groups of 50, 60, and 70 years of age between CH and SNT. The expression of miRNA was different between SNT and HCC only in patients in their 70s. Of the 55 miRNAs with significant differences in expression between CH and SNT, 34 miRNAs showed significant differences in expression even in the degree of liver fibrosis. The observation that miRNAs involved in hepatocarcinogenesis differ at different ages suggests that the mechanisms of carcinogenesis differ by age group as well. We also found that many miRNAs whose expression did not affect liver fibrosis were involved in carcinogenesis. These findings are expected to define biomarkers for detection of HCC at early stage, and develop novel therapeutic targets for HCC.

## 1. Introduction

Hepatocellular carcinoma (HCC)-related deaths (2018) are estimated to be the second highest in men, the sixth highest in women, and the fourth highest overall among all cancers worldwide [1]. High risk factors for HCC include cirrhosis and age [2]. Importantly, elderly patients have been reported to develop HCC even without significant liver fibrosis, suggesting that aging itself may predispose individuals to hepatocarcinogenesis [3]. Like in many age-related degenerative diseases, the incidence of cancer is known to increase almost exponentially beginning from 50 to 60 years of age, about the midpoint of a person’s lifespan [4,5]. The eradication of the virus has been suggested to be more effective in preventing HCC in young patients chronically infected with hepatitis C virus (HCV) than in older patients [6]. Aging causes characteristic changes in cells and tissues due to a progressive decline in function. Liver volume and perfusion decrease markedly with age [7]. The regenerative capacity of the liver also declines with age [8,9].

MicroRNAs (miRNAs) are non-coding RNAs approximately 20 base pairs in length that regulate target gene expression in a nucleotide sequence-specific manner. Aberrant miRNA expression has been reported to be associated with carcinogenesis [10,11].

Studies of miRNAs involved in aging are being vigorously conducted using *Caenorhabditis elegans* (*C. elegans*) model [12,13,14]. The expression pattern of let-7 and miR-34 family related to aging is known to be well correlated between human and *C. elegans* [15]. In mice, miRNA studies in liver aging showed that the overexpression of miR-34a, miR-93, miR-214, miR-669c, and miR-709 was correlated with liver aging through effects on glutathione-S-transferase, Silent information regulator 2 homolog 1 (SIRT1), Specificity protein 1 (SP1), and Nuclear Factor (Erythroid-Derived 2)-Like 2 (NRF2) [16,17]. The overexpression of 10 miRNAs, including miR-27a, is associated with increased survival [18]. Studies in mice have shown that reduced levels of Insulin-like Growth Factor-I (IGF1) plays a role in the aging of hematopoietic stem cells [19], and that IGF1 is regulated by miR-1 in a mouse model of progeria [20].

miRNA expression in human HCC has been comprehensively analyzed using next-generation sequencing. Compared to normal liver, 86% of miRNAs were downregulated, 13% were normally expressed, and less than 1% were upregulated in HCC [21]. Approximately 52% of the miRNAs expressed in liver tissue were miR-122, followed by 16.9% miR-192 and 4.9% miR-199-a/b-3p [22,23,24]. The expression levels of miR-92, 20, and 18 were inversely correlated with the degree of HCC differentiation [25], and that of miR-18b was directly correlated with the prognosis and degree of cancer differentiation [26]. The miRNA profile in surrounding HCC tissues reflected the genomic accumulation of aberrations associated with multicentric de novo carcinogenesis [27]. In the same study that assessed HCC recurrence risk after hepatic resection based on miRNA expression profiling, tumor miRNA profiles predicted early recurrence and non-tumor tissue miRNA profiles predicted late recurrence [27]. These studies suggest that miRNA profiles can predict HCC malignancy.

In this study, we established miRNA expression patterns in a total of nine groups of chronic hepatitis, cancer, and non-cancer patients in their 50s, 60s, and 70s, with the aim being to clarify whether miRNAs involved in carcinogenesis show different expression pattern by age.

## 2. Results

### 2.1. Clinical Characteristics of Each Disease Group by Age

In CH, two age groups were randomly selected and compared for a total of 13 parameters including 11 blood test factors, the degree of hepatitis, and the stage of liver fibrosis. In the comparison between the under-50s group and other groups, statistical significance was found in each item of Alanine transaminase (ALT), white blood cell (WBC), Alkaline phosphatase (ALP), Gamma-glutamyltransferase (GGTP), and Albumin (Alb), but in the comparison between the under-50s, -60s, and -70s groups, some of the factors, such as ALT and Alb, showed significant differences. A histopathological analysis revealed a statistically significant difference between the group under 50 years old and the other groups. Four parameters in HCC, namely two blood test factors, the stage of HCC, and the histological difference, and one parameter in SNT, namely the stage of liver fibrosis, were analyzed by age group, but these parameters showed no statistically significant differences (Table 1 and Supplementary Tables S1 and S2).

### 2.2. Differences between Age Groups in the Same Disease

**Figure 1:**
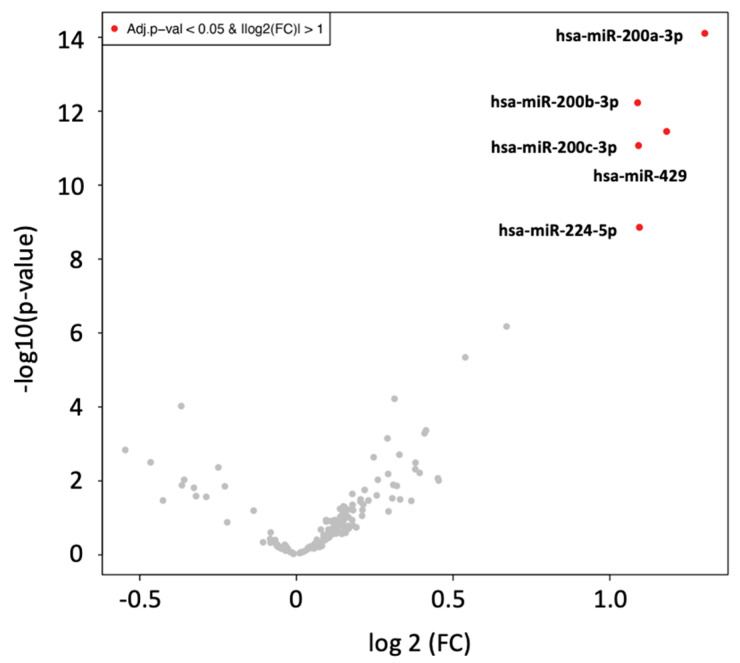
miRNAs differentially expressed with age. A volcano plot shows miRNAs with different expression levels when CH is divided by 50 years of age. miRNAs with adjusted *p-*values 0.05 and expression ratios >2 folds in both groups (red dots). miRNAs with no significant difference in expression are indicated by gray dots.CH, chronic hepatitis C; miRNA, microRNA.

We classified the ages of CH, HCC, and SNT into three groups, namely patients in their 50s, 60s, and 70s, and attempted to identify miRNAs with differential expression depending on age. A specific miRNA expression pattern in each age group could not be identified in three disease groups (Supplementary Figure S1 and Supplementary Table S3). We then attempted to identify miRNAs whose expression was altered in patients aged under 50 years of age and those 50 years old or older (years: 50s, 60s, 70s). The expression of five miRNAs (hsa-miR-200a-3p, 200b-3p, 200c-3p, 224-5p, and 429) was significantly elevated in the group ≥50 years old (Figure 1).

### 2.3. Differences between Diseases in the Same Age Group

**Figure 2:**
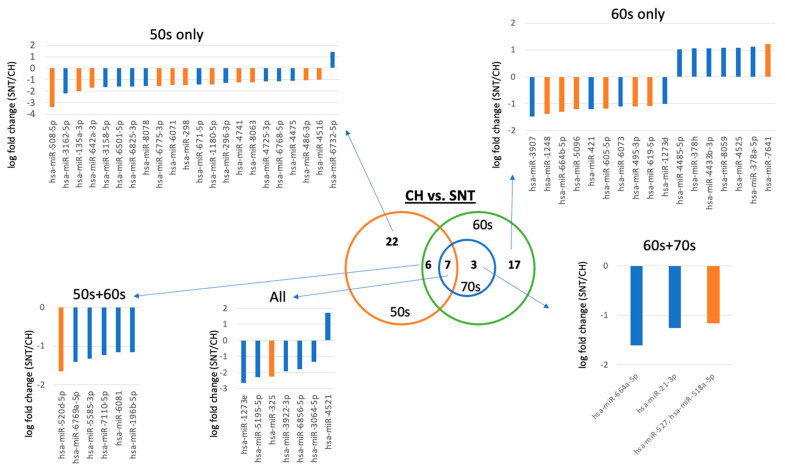
Comparison of miRNA expression between CH and SNT. Venn diagrams show the number of miRNAs with different expression levels in each age group. In addition, the relative expression of miRNAs by age is shown using bar graphs. Orange bars indicate cases where oncogenic potential and expression patterns are consistent, for example, when the expression of miRNA related to cancer suppression is suppressed in SNT compared to CH, and when the expression of miRNA related to cancer promotion is enhanced in SNT compared to CH. CH, chronic hepatitis C; miRNA, microRNA; SNT, surrounding non-cancerous tissues.

Since no differences were found between age groups for the same disease, differences between disease groups were evaluated by age. Comparing CH and SNT, the expression level of 55 miRNAs was significantly different (Figure 2 and Supplementary Figure S2).

Seven miRNAs were differentially expressed across all age groups. Among them, the expression level of six miRNAs (hsa-miR-1273e, 5195-5p, 325, 3922-3p, 6856-5p, and 3064-5p) was decreased in SNT, whereas the expression level of miR-4521 was increased in SNT. An evaluation of previously reported miRNAs based on their functions shows that hsa-miR-325 has carcinogenic potential, which is consistent with the higher carcinogenic potential in SNT compared to CH (Table 2 and Supplementary Table S4). There were six different miRNAs whose expression pattern was the same expression pattern between patients in their 50s and 60s, and the expression of all six miRNAs was suppressed in SNT compared to CH. Here, the expression of miR-520s-5p, which has oncogenic potential, was increased in SNT. There were three miRNAs whose expression was the same expression pattern between patients in their 60s and 70s, and the expression of all miRNAs was suppressed in SNT compared to CH. In this group, the expression of miR-518a-5p/527, which has oncogenic potential, was increased in SNT. There were 22 miRNAs whose expression was different only in the 50s age group, 21 of which were downregulated in SNT and 1 which was upregulated in SNT compared to CH. Here, the expression of 11 miRNAs, which has oncogenic potential, was increased in SNT. There were 17 miRNAs whose expression was different only in the 60s age group, 10 miRNAs whose expression was suppressed in SNT, and 7 miRNAs whose expression was increased in SNT. There was no miRNA whose expression was changed only at 70 years old. In this group, the expression of miR-7641, which has tumorigenic potential, was suppressed by SNT, and the expression of six miRNAs, all of which have tumorigenic potential, was increased in SNT (Figure 2, Supplementary Figure S2, Table 2, and Supplementary Table S4).

**Figure 3:**
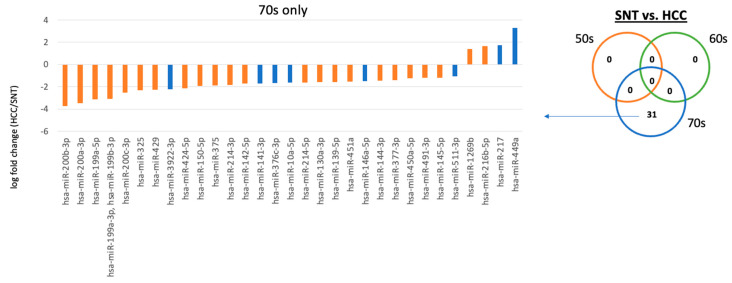
Comparison of miRNA expression between SNT and HCC. Relative expression ratios of miRNAs in each age group are shown. Venn diagrams show the number of miRNAs with different expression for each age group. In addition, the relative expression of miRNAs by age is shown in bar graphs. Orange bars indicate cases where oncogenic potential and expression patterns are consistent. HCC, hepatocellular carcinoma; miRNA, microRNA; SNT, surrounding non tumorous tissues.

When comparing SNT and HCC, there were miRNAs that showed differences in expression only at the age of 70 years. Thirty-one miRNAs were differentially expressed between SNT and HCC, 27 of which were downregulated in HCC and 4 upregulated in HCC at 70 years old. At 50 and 60 years old, miRNAs whose expression was significantly different between the two groups could not be identified. Among these, the expression of 21 miRNAs with oncogenic potential was suppressed in HCC, and the expression of 2 miRNAs with oncogenic potential was upregulated in HCC (Figure 3, Supplementary Figure S2, Table 2, and Supplementary Table S4).

### 2.4. miRNAs Affected by the Stage of Liver Fibrosis

**Figure 4:**
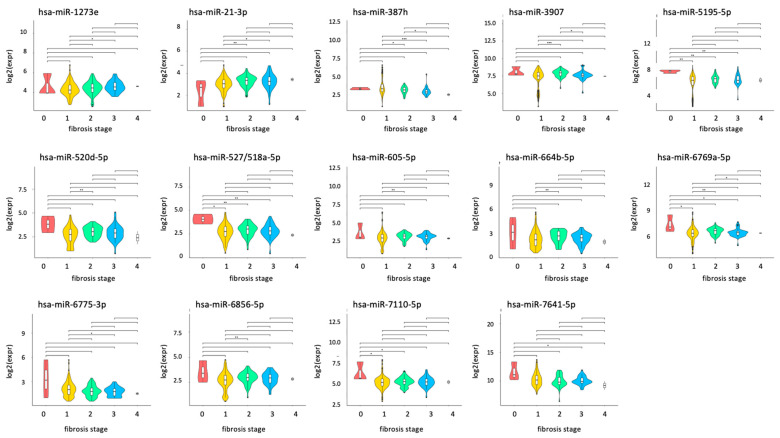
Comparison of miRNA expression according to fibrosis stages. Significant differences in miRNA expression between the two groups at different stages of liver fibrosis are shown. Asterisk indicates the significant difference. miRNA, microRNA. *: *p* value < 0.05, **: *p* value < 0.01, ***: *p* value < 0.001

As shown in previous section, there were 55 miRNAs with significantly different expression levels between CH and SNT. We examined whether there was a relationship between the expression level of these miRNAs and stage of liver fibrosis. The expression level of 14 miRNAs (hsa-miR-1273e, 21-3p, 378h, 3907, 5195-5p, 520d-5p, 527/518a-5p, 605-5p, 664b-5p, 6769a-5p, 6775-3p, 6856-5p, 7110-5p, and 7641) was significantly different between two fibrosis stages (Figure 4 and Supplementary Table S5). In contrast, there was no significant difference in the expression of 41 miRNAs among different stages.

### 2.5. Disease Classification Using Random Forest

**Figure 5:**
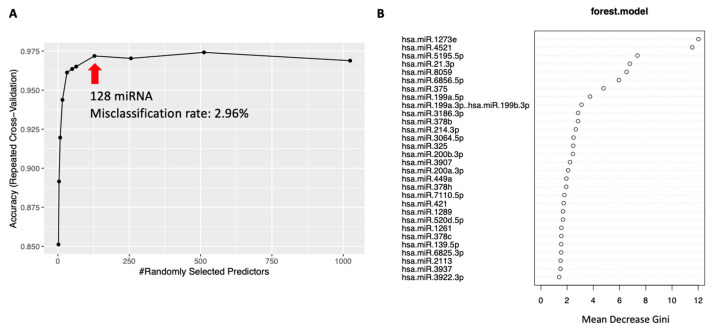
Disease classification using random forest. (**A**) Relationship between the number of miRNAs used to classify diseases and the correct response rate for disease diagnosis. When classifying using 128 miRNAs, the misclassification rate is 2.96%. (**B**) The top 30 miRNAs contributing to classification are listed using the Mean Decrease Gini. miRNA, microRNA.

miRNAs that contributed to the classification were retrieved in order of importance. Analysis was performed using all cases without division by age. Using information on 128 miRNAs, the misclassification rate for all diseases was 2.96% (Figure 5A). The top 30 miRNAs with high importance are shown in Mean Decrease in Gini (Figure 5B). Among these, 14 miRNAs were expressed differentially depending on the degree of liver fibrosis, and 8 were differentially expressed showing significant differences.

## 3. Discussion

To date, most miRNA analyses involved in HCC have compared cancer and non-cancer tissues to identify miRNAs whose expression is altered in cancer tissues. This research focuses on the following two points, which is different from the conventional research. First, we analyzed miRNA expression patterns in three different age cohorts separately, because the incidence of HCC generally increases from the age of 60 years [4,5]. Second, liver tissue after cancer treatment is known to have a high carcinogenic potential [28,29]; however, the carcinogenic effects of surrounding tissues have not been well analyzed. We generated miRNA expression profiles for patient cohorts in their 50s, 60s, and 70s, segmented by a 10-year age group for cancer tissue, surrounding non-cancer tissue, and chronic hepatitis tissue. Furthermore, in the CH cohort, we also compared the miRNA expression profiles between patients under and over 50 years of age.

We examined whether there were differences in clinical data among different age groups in each disease. No differences were observed in HCC and SNT between the age groups 50s, 60s, and 70s. Differences in CH were observed in the group <50 years old. Similarly, no difference in miRNA expression was observed between the age groups 50s, 60s, and 70s in HCC and SNT, whereas some miRNAs in the CH group showed differences in expression between the age groups <50 and ≥50 years old. Although miRNAs whose expression changes in association with aging are known [30], no significant difference in their expression was observed between HCC and SNT.

First, when comparing CH and SNT, we found that the expression of many miRNAs differed in participants in their 50s. Compared to CH, where no cancer tissue exists, SNT adjacent to cancer has a higher expression of miRNAs that have been reported to suppress carcinogenesis. miRNAs whose expression is suppressed by SNT even in the 60s age group have been reported to have cancer-suppressive functions. In particular, the expression level of miR-325 is suppressed in SNT compared to CH and miR-325 has been reported as anti-oncomiR (anti-oncogenic miRNA) in HCC [31]. It was also reported that the expression of miR-520d-5p as anti-oncomiR [32] was suppressed in both the 50s and 60s age groups, and that of miR-527/miR-518a-5p is also known as anti-oncomiR [33] was suppressed in SNT in both the 60s and 70s age groups. These expression patterns suggest that when the expression of these miRNAs is not suppressed by CH, hepatocarcinogenesis does not occur, but the oncogenic potential is enhanced when the expression is suppressed under some circumstances. In contrast, in each age group, the expression of 11 miRNAs (Supplementary Table S3) with tumor suppressive effects was suppressed in the 50s age group, the expression of 7 miRNAs with tumor suppressive effects was suppressed in the 60s age group, and the oncogenic miR-7641 was upregulated in SNT [34]. There were no miRNAs with characteristic expression in both CH and SNT at 70 years old. The expression of miRNAs with tumor-suppressive effects was suppressed in each age group, and different miRNAs with tumor-suppressive effects were involved in the enhancement of carcinogenic potential in each age group. Next, when comparing SNT and HCC, there was no miRNA that showed a difference in expression between 50s and 60 years. However, in the 70 years group, the expression of many miRNAs was suppressed in HCC, and many of these miRNAs were reported to have tumor suppressive functions. In SNT and HCC, unlike CH and SNT, 31 miRNAs with abberant expression specific to the 70s age group were identified, 21 of which had tumor-suppressive effects, and these miRNAs were downregulated in HCC (Supplementary Table S3). In addition, two miRNAs (hsa-miR-1269b and 216b-5p) have cancer-promoting effects, and the expression of these miRNAs was enhanced in HCC. In summary, in comparing between CH and SNT, and between SNT and HCC, hepatocarcinogenesis can be associated with the suppressed expression of miRNAs that have cancer-suppressive functions. Although the types of miRNAs involved in carcinogenesis are different, an analysis by age shows that in the 50s and 60s age groups, the tumorigenic potential increases as a result of suppressing the tumor suppressor miRNA in SNT, and in the 70s age groups, the tumor growth potential increases as a result of inhibiting the tumor suppressor miRNAs in HCC.

Hepatocarcinogenesis is closely related to aging and liver fibrosis. Therefore, it was assumed that miRNAs related to aging were also involved in disease differentiation. However, there were no miRNAs that showed differences in expression depending on age in each disease group (Supplementary Table S1), and there were miRNAs that showed differences in expression only in the analysis between diseases. It was estimated that age was not relevant as a confounding factor when classifying diseases (Figure 2, Figure 3, and Supplementary Figure S1). Furthermore, we selected the top 30 miRNAs contributing to disease classification using random forest and compared them with miRNAs whose expression differed depending on the degree of fibrosis. Eight mRNAs were found to be common to disease classification and fibrosis classification. Based on these results, we concluded that the changes in miRNA used to distinguish between diseases are not significantly influenced by the degree of liver fibrosis.

Finally, we investigated the effect of sample collection method on miRNA expression. In examining whether gene expression profiles (GEPs) detected in tumor biopsies in breast cancer subjects are representative of the whole tumor, GEPs detected in core biopsies correlate very well with GEPs in surgical samples (rs ≥ 0.95, *p* < 0.001) [35]. In addition, in determining the treatment strategy for breast cancer, there is a study comparing the estrogen (ER), progesterone (PR), and HER2 levels in tissues obtained using needle biopsy and surgical resected tissues (n = 916; 94.8%), PR (n = 1170; 86.7%), and HER2 (n = 881; 98.1%), with very low discordance between ER/PR/HER2 test results. It has been reported that the concordance rate is particularly high in patients who did not undergo neoadjuvant chemotherapy. Since our analysis targets HCC and CH that have not undergone preoperative chemotherapy including anti-viral therapy, it is expected that the discrepancy rate in gene carcinogenesis analysis results in tissues obtained by needle biopsy and excision will be low [36].

We believe that this analysis has limitations in the following respects. This analysis did not observe the same patients over time, but rather compared patients of the same age. In addition, the ratio of the number of patients with chronic hepatitis analyzed to the number of patients with cancer analyzed is quite large. In addition, this is an observational study, and it is difficult to reproduce these phenomena in cell lines or experimental animals.

## 4. Materials and Methods

### 4.1. Analysis Strategy

Group definition: CH group included participants prior to antiviral treatment, who revealed no HCC in blood tests and imaging tests. Liver tissues of 360 participants were isolated using needle biopsy, liver inflammation and fibrosis were evaluated using hematoxylin and eosin (HE) staining, and blood was collected at the same time as the needle biopsy. In the HCC group, the tissues were obtained during surgical resection in 43 cases. The cancer stage was determined by imaging tests before surgery, and blood samples were obtained before surgery. Non-tumor tissue surrounding hepatocellular carcinoma (SNT) samples refer to the non-cancerous tissue surrounding a tumor in HCC, and the tissue was obtained during surgical resection in 43 cases in HCC group. The stage of liver fibrosis was evaluated using HE staining. Cases were further divided into the following age groups: 50s (50–59), 60s (60–69), and 70s (70–79). In addition, only CH cases under 50 years of age were included in the analysis (Table 3, Supplementary Tables S6 and S7).

### 4.2. RNA Preparation and Microarray Analysis

Microarray analysis was performed using total RNA derived from 360 samples of CH, 43 of HCC, and 43 of SNT. HCC and SNT were resected, and CH was obtained by needle biopsy. After collection, the liver tissue was immediately frozen in liquid nitrogen. Total RNA from tissue samples was extracted using a mirVana miRNA extraction Kit (Thermo Fisher Scientific, Waltham, MA, USA) according to the manufacturer’s instructions. Overall, 100 ng of total RNA was analyzed using 3D-Gene miRNA microarray (Toray Industries, Inc., Kanagawa, Japan). Comprehensive miRNA expression analysis was performed using a 3D-Gene miRNA Labeling Kit and a 3D-Gene Human miRNA Oligo Chip (Toray Industries, Inc.), both of which could detect 2555 miRNA sequences in miRBase release 20 (http://www.mirbase.org/ (accessed on 25 Mar 2020)). All microarray datasets from this study conform to “Minimum Information About a Microarray Experiment” guidelines and are publicly available in the GEO database [(GSE147892 (CH), GSE147889 (HCC), GSE147887 (SNT)].

### 4.3. Statistical Analysis

#### 4.3.1. Clinical Data Comparison among Groups

Arbitrary comparisons of clinical data among any two groups including 11 blood examination items (CH), 2 blood examination items (HCC), HCC stage, histological differentiation (HCC), grade of liver inflammation (CH), and stage of liver fibrosis (CH and SNT)) were performed using Student’s *t*-test.

#### 4.3.2. Analysis of Differences in miRNA between Age Groups for the Same Disease

To preprocess the microarray data, expression levels of miRNA in each sample were scaled by log2. Comparisons between age groups were performed using the Kruskal–Wallis test for CH, HCC, and SNT. Three age groups were as follows: 50s, 60s, and 70s. *p* and *q* values for each miRNA were calculated using the Benjamini–Hochberg (BH) method. miRNAs with a *q* value < 0.05 (FDR < 0.05) and an expression variation value (FC) of 2 times or higher were considered different between different age groups.

#### 4.3.3. Analysis of Differences between Diseases in the Same Age Group

By comparing CH and HCC, CH and SNT, and HCC and SNT in each age group, we detected differentially expressed miRNAs. Three age groups were used: 50s, 60s, and 70s. The linear model used the method “limma”, which was also based on a *t*-test [37]. The *p* value of the obtained miRNA was corrected using the BH method and the *q* value was calculated. The criteria for extracting miRNAs with differences between disease groups were the same as in the previous analysis.

#### 4.3.4. Analysis of miRNAs Involved in Liver Fibrosis

A significance test (Wilcoxon rank sum test) was performed using miRNAs with significant differences in expression between CH and SNT (Supplementary Table S3).

#### 4.3.5. Evaluation of Factors That Contributed to Classification when Performing Group Classification

Disease classification was performed using Random Forest. The analysis procedure is shown. After parameter tuning using cross-validation, classification was performed using the optimal model. miRNAs that contributed to the classification were retrieved in order of importance.

## 5. Conclusions

In this analysis, no miRNAs showed changes in expression with age in either of the conditions tested; however, there were miRNAs whose expression was altered with age among diseases. In particular, in the 50s and 60s age groups, miRNAs with a tumor suppressor function were suppressed in SNT, and in the 70s age group, miRNAs with a tumor suppressor function were suppressed in HCC, suggesting that the carcinogenesis mode differed by age. Furthermore, although carcinogenesis and the progression of aging and fibrosis are closely related, it has become clear that they show independent miRNA expression patterns. This information is based on clinical material and has not been verified by in vitro/in vivo studies. In order to utilize these as new cancer prediction markers and therapeutic targets, it is expected that more important miRNAs will be narrowed down and verification experiments will be performed to make them clinically useful.

## Figures and Tables

**Table 1:** Summary of clinical data by disease and age group (CH group).

Group According to Age (Years)	Unit	<50	50s	60s	70s
histology (A)		1.20 ± 0.51	1.54 ± 0.64	1.59 ± 0.72	1.70 ± 0.61
histology (F)		1.10 ± 0.58	1.45 ± 0.74	1.60 ± 0.84	1.67 ± 0.68
HCVRNA	log copies/mL	6.15 ± 1.03	6.07 ± 0.81	6.00 ± 0.90	5.97 ± 0.78
AST	IU/L	48.02 ± 46.30	57.14 ± 39.59	50.75 ± 32.06	63.07 ± 44.93
ALT	IU/L	68.3 ± 73.50	71.58 ± 61.95	52.91 ± 37.04	44.92 ± 75.30
WBC	μL	6000 ± 1720	5440 ± 1680	5110 ± 1250	4890 ± 1190
PLT	x10^4/μL	23.48 ± 5.80	19.25 ± 6.42	16.82 ± 5.19	17.35 ± 4.92
T_BIL	mg/dL	0.69 ± 0.32	0.73 ± 0.33	0.70 ± 0.26	0.63 ± 0.18
ALP	IU/L	231.41 ± 97.09	273.54 ± 110.06	301.44 ± 131.39	289.00 ± 90.63
GGTP	U/L	75.68 ± 122.54	60.18 ± 60.20	50.66 ± 64.45	61.07 ± 62.70
Hb	g/dL	14.08 ± 1.83	13.95 ± 1.35	13.74 ± 1.33	13.82 ± 1.06
ALB	g/dL	4.41 ± 0.34	4.28 ± 0.33	4.16 ± 0.35	4.25 ± 0.32
AFP	ng/mL	3.06 ± 7.41	4.41 ± 6.06	5.75 ± 8.90	6.40 ± 7.75
Summary of clinical data by disease and age group (HCC and SNT group)					
Group according to age (years)	unit	50s	60s	70s	
Stage		1.50 ± 0.71	1.82 ± 0.40	2.11 ± 0.75	
AFP	ng/ml	26.30 ± 20.02	129.38 ± 199.08	37.77 ± 83.58	
DCP	U/ml	6940 ± 12,000	398.1 ± 990.0	3670 ± 13,100	
differentiation (T)		2.00 ± 0.00	1.83 ± 0.58	1.64 ± 0.49	
fibrosis (N)		3.33 ± 0.58	3.38 ± 0.52	3.36 ± 0.49	

Abbreviations: METAVIR SCORE was used for CH pathological histology {Bedossa et al., Hepatology, 1996}; T indicates HCC; N indicates SNT; DCP: des gamma carboxy prothrombin.

**Table 2:** List of previously reported effects of miRNAs on carcinogenesis that show differences in expression between each disease.

Age Group (Years)	Comparison	miRNA Function	
		tumor suppressor	oncogene
50s	CH vs. SNT	hsa-miR-1180-5p	hsa-miR-296-3p
		hsa-miR-135a-3p	hsa-miR-671-5p
		hsa-miR-298	
		hsa-miR-4516	
		hsa-miR-4741	
		hsa-miR-486-3p	
		hsa-miR-508-5p	
		hsa-miR-6071	
		hsa-miR-642a-3p	
		hsa-miR-6775-3p	
		hsa-miR-8063	
50s_60s	CH vs. SNT	hsa-miR-520d-5p	hsa-miR-196b-5p
50s_60s_70s	CH vs. SNT	hsa-miR-325	hsa-miR-3064-5p
		hsa-miR-4521	
60s	CH vs. SNT	hsa-miR-1248	hsa-miR-3907
		hsa-miR-378a-5p	hsa-miR-421
		hsa-miR-495-3p	hsa-miR-7641
		hsa-miR-5096	
		hsa-miR-605-5p	
		hsa-miR-619-5p	
		hsa-miR-664b-5p	
60s_70s	CH vs. SNT	hsa-miR-527, hsa-miR-518a-5p	hsa-miR-21-3p
70s	SNT vs. HCC	hsa-miR-217	hsa-miR-1269b
		hsa-miR-449a	hsa-miR-216b-5p
		hsa-miR-130a-3p	hsa-miR-10a-5p
		hsa-miR-139-5p	hsa-miR-141-3p
		hsa-miR-142-5p	hsa-miR-146a-5p
		hsa-miR-144-3p	hsa-miR-376c-3p
		hsa-miR-145-5p	
		hsa-miR-150-5p	
		hsa-miR-199a-3p, hsa-miR-199b-3p	
		hsa-miR-199a-5p	
		hsa-miR-200a-3p	
		hsa-miR-200b-3p	
		hsa-miR-200c-3p	
		hsa-miR-214-3p	
		hsa-miR-214-5p	
		hsa-miR-325	
		hsa-miR-375	
		hsa-miR-377-3p	
		hsa-miR-424-5p	
		hsa-miR-429	
		hsa-miR-450a-5p	
		hsa-miR-451a	

**Table 3:** Age composition of each disease group.

Age (Years)	CH	HCC	SNT	Subtotal
50>	93	0	0	93
51–60	115	3	3	118
61–70	134	12	12	146
71–80	18	28	28	46
Total	360	43	43	403

Abbreviations: CH, chronic hepatitis C; HCC, hepatocellular carcinoma; SNT, surrounding non-cancerous tissues.

## Data Availability

All microarray datasets from this study conform to “Minimum Information About a Microarray Experiment” guidelines and are publicly available in the GEO database [(GSE147892 (CH), GSE147889 (HCC), GSE147887 (SNT)].

## Supplementary Materials

The following supporting information can be downloaded at: https://www.mdpi.com/article/10.3390/ijms25147858/s1.

## References

[1] Bray F., Ferlay J., Soerjomataram I., Siegel R.L., Torre L.A., Jemal A. (2018). Global cancer statistics 2018: GLOBOCAN estimates of incidence and mortality worldwide for 36 cancers in 185 countries. CA Cancer J. Clin..

[2] Altekruse S.F., McGlynn K.A., Reichman M.E. (2009). Hepatocellular carcinoma incidence, mortality, and survival trends in the United States from 1975 to 2005. J. Clin. Oncol..

[3] Honda T., Miyaaki H., Ichikawa T., Taura N., Miuma S., Shibata H., Isomoto H., Takeshima F., Nakao K. (2011). Clinical characteristics of hepatocellular carcinoma in elderly patients. Oncol. Lett..

[4] Balducci L., Ershler W.B. (2005). Cancer and ageing: A nexus at several levels. Nat. Rev. Cancer.

[5] Jemal A., Siegel R., Xu J., Ward E. (2010). Cancer statistics, 2010. CA Cancer J. Clin..

[6] Asahina Y., Tsuchiya K., Tamaki N., Hirayama I., Tanaka T., Sato M., Yasui Y., Hosokawa T., Ueda K., Kuzuya T. (2010). Effect of aging on risk for hepatocellular carcinoma in chronic hepatitis C virus infection. Hepatology.

[7] Wynne H.A., Cope L.H., Mutch E., Rawlins M.D., Woodhouse K.W., James O.F. (1989). The effect of age upon liver volume and apparent liver blood flow in healthy man. Hepatology.

[8] Roberts C.J., Jackson L., Halliwell M., Branch R.A. (1976). The relationship between liver volume, antipyrine clearance and indocyanine green clearance before and after phenobarbitone administration in man. Br. J. Clin. Pharmacol..

[9] Caesar J., Shaldon S., Chiandussi L., Guevara L., Sherlock S. (1961). The use of indocyanine green in the measurement of hepatic blood flow and as a test of hepatic function. Clin. Sci..

[10] Rupaimoole R., Slack F.J. (2017). MicroRNA therapeutics: Towards a new era for the management of cancer and other diseases. Nat. Rev. Drug Discov..

[11] Tutar Y. (2014). miRNA and cancer; computational and experimental approaches. Curr. Pharm. Biotechnol..

[12] Ibanez-Ventoso C., Yang M., Guo S., Robins H., Padgett R.W., Driscoll M. (2006). Modulated microRNA expression during adult lifespan in Caenorhabditis elegans. Aging Cell.

[13] de Lencastre A., Pincus Z., Zhou K., Kato M., Lee S.S., Slack F.J. (2010). MicroRNAs both promote and antagonize longevity in *C*. elegans. Curr. Biol..

[14] Kato M., Chen X., Inukai S., Zhao H., Slack F.J. (2011). Age-associated changes in expression of small, noncoding RNAs, including microRNAs, in *C*. elegans. RNA.

[15] Ibanez-Ventoso C., Vora M., Driscoll M. (2008). Sequence relationships among C. elegans, D. melanogaster and human microRNAs highlight the extensive conservation of microRNAs in biology. PLoS ONE.

[16] Maes O.C., An J., Sarojini H., Wang E. (2008). Murine microRNAs implicated in liver functions and aging process. Mech. Ageing Dev..

[17] Li N., Muthusamy S., Liang R., Sarojini H., Wang E. (2011). Increased expression of miR-34a and miR-93 in rat liver during aging, and their impact on the expression of Mgst1 and Sirt1. Mech. Ageing Dev..

[18] Bates D.J., Li N., Liang R., Sarojini H., An J., Masternak M.M., Bartke A., Wang E. (2010). MicroRNA regulation in Ames dwarf mouse liver may contribute to delayed aging. Aging Cell.

[19] Young K., Eudy E., Bell R., Loberg M.A., Stearns T., Sharma D., Velten L., Haas S., Filippi M.D., Trowbridge J.J. (2021). Decline in IGF1 in the bone marrow microenvironment initiates hematopoietic stem cell aging. Cell Stem Cell.

[20] Marino G., Ugalde A.P., Fernandez A.F., Osorio F.G., Fueyo A., Freije J.M., Lopez-Otin C. (2010). Insulin-like growth factor 1 treatment extends longevity in a mouse model of human premature aging by restoring somatotroph axis function. Proc. Natl. Acad. Sci. USA.

[21] Hou J., Lin L., Zhou W., Wang Z., Ding G., Dong Q., Qin L., Wu X., Zheng Y., Yang Y. (2011). Identification of miRNomes in human liver and hepatocellular carcinoma reveals miR-199a/b-3p as therapeutic target for hepatocellular carcinoma. Cancer Cell.

[22] Kutay H., Bai S., Datta J., Motiwala T., Pogribny I., Frankel W., Jacob S.T., Ghoshal K. (2006). Downregulation of miR-122 in the rodent and human hepatocellular carcinomas. J. Cell Biochem..

[23] Gramantieri L., Ferracin M., Fornari F., Veronese A., Sabbioni S., Liu C.G., Calin G.A., Giovannini C., Ferrazzi E., Grazi G.L. (2007). Cyclin G1 is a target of miR-122a, a microRNA frequently down-regulated in human hepatocellular carcinoma. Cancer Res..

[24] Coulouarn C., Factor V.M., Andersen J.B., Durkin M.E., Thorgeirsson S.S. (2009). Loss of miR-122 expression in liver cancer correlates with suppression of the hepatic phenotype and gain of metastatic properties. Oncogene.

[25] Murakami Y., Yasuda T., Saigo K., Urashima T., Toyoda H., Okanoue T., Shimotohno K. (2006). Comprehensive analysis of microRNA expression patterns in hepatocellular carcinoma and non-tumorous tissues. Oncogene.

[26] Murakami Y., Tamori A., Itami S., Tanahashi T., Toyoda H., Tanaka M., Wu W., Brojigin N., Kaneoka Y., Maeda A. (2013). The expression level of miR-18b in hepatocellular carcinoma is associated with the grade of malignancy and prognosis. BMC Cancer.

[27] Sato F., Hatano E., Kitamura K., Myomoto A., Fujiwara T., Takizawa S., Tsuchiya S., Tsujimoto G., Uemoto S., Shimizu K. (2011). MicroRNA profile predicts recurrence after resection in patients with hepatocellular carcinoma within the Milan Criteria. PLoS ONE.

[28] Yoshida H., Shiratori Y., Moriyama M., Arakawa Y., Ide T., Sata M., Inoue O., Yano M., Tanaka M., Fujiyama S. (1999). Interferon therapy reduces the risk for hepatocellular carcinoma: National surveillance program of cirrhotic and noncirrhotic patients with chronic hepatitis C in Japan. IHIT Study Group. Inhibition of Hepatocarcinogenesis by Interferon Therapy. Ann. Intern. Med..

[29] Tateishi R., Shiina S., Yoshida H., Teratani T., Obi S., Yamashiki N., Yoshida H., Akamatsu M., Kawabe T., Omata M. (2006). Prediction of recurrence of hepatocellular carcinoma after curative ablation using three tumor markers. Hepatology.

[30] Ghafouri-Fard S., Abak A., Talebi S.F., Shoorei H., Branicki W., Taheri M., Akbari Dilmaghani N. (2021). Role of miRNA and lncRNAs in organ fibrosis and aging. Biomed. Pharmacother..

[31] Li L., Ji Y., Chen Y.C., Zhen Z.J. (2021). MiR-325-3p mediate the CXCL17/CXCR8 axis to regulate angiogenesis in hepatocellular carcinoma. Cytokine.

[32] Liu S., Bu X., Kan A., Luo L., Xu Y., Chen H., Lin X., Lai Z., Wen D., Huang L. (2022). SP1-induced lncRNA DUBR promotes stemness and oxaliplatin resistance of hepatocellular carcinoma via E2F1-CIP2A feedback. Cancer Lett..

[33] Nomura K., Kitanaka A., Iwama H., Tani J., Nomura T., Nakahara M., Ohura K., Tadokoro T., Fujita K., Mimura S. (2021). Association between microRNA-527 and glypican-3 in hepatocellular carcinoma. Oncol. Lett..

[34] Kumar M., Kaur R., Kanthaje S., Dhiman R.K., Chakraborti A. (2022). Bacterial metabolite butyrate in modulating sorafenib-targeted microRNAs to curtail its resistance in hepatocellular carcinoma. J. Cancer Res. Clin. Oncol..

[35] Zanetti-Dallenbach R., Vuaroqueaux V., Wight E., Labuhn M., Singer G., Urban P., Eppenberger U., Holzgreve W., Eppenberger-Castori S. (2006). Comparison of gene expression profiles in core biopsies and corresponding surgical breast cancer samples. Breast Cancer Res..

[36] Berghuis A.M.S., van Deurzen C.H.M., Koffijberg H., Terstappen L., Sleijfer S., IJzerman I.J. (2019). Real-world data on discordance between estrogen, progesterone, and HER2 receptor expression on diagnostic tumor biopsy versus tumor resection material. Breast Cancer Res. Treat..

[37] Ritchie M.E., Phipson B., Wu D., Hu Y., Law C.W., Shi W., Smyth G.K. (2015). Limma powers differential expression analyses for RNA-sequencing and microarray studies. Nucleic Acids Res..

