# Supplementary figures and images for: Analysis of Carcinogenic Involvement of MicroRNA Pattern in Peripheral Non-Cancerous Tissues and Chronic Viral Liver Injury
Source: Int J Mol Sci. 2024 Jul 18;25(14):7858. doi: 10.3390/ijms25147858 (PMC11277156; doi:10.3390/ijms25147858)

**A**

50s

60s

70s

All ages

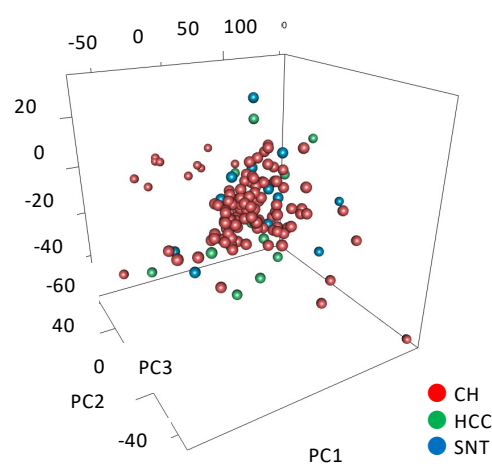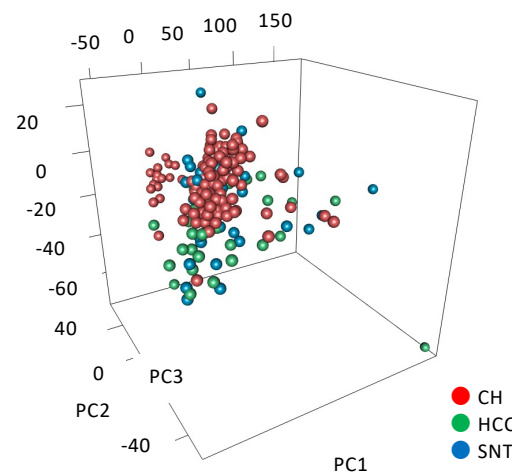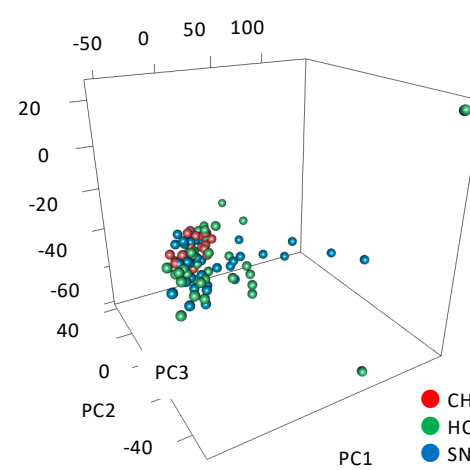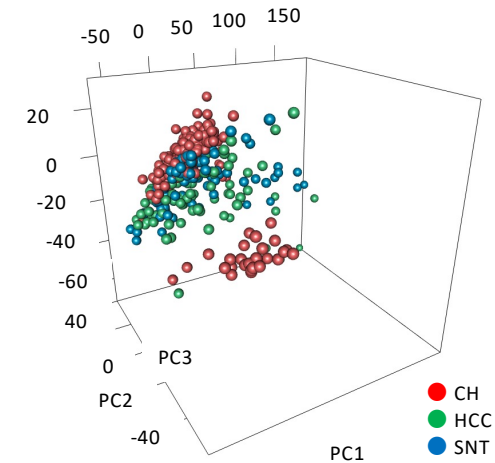**B**

CH

SNT

HCC

All disease

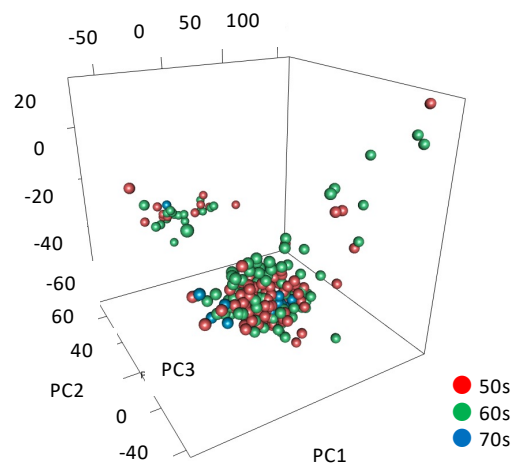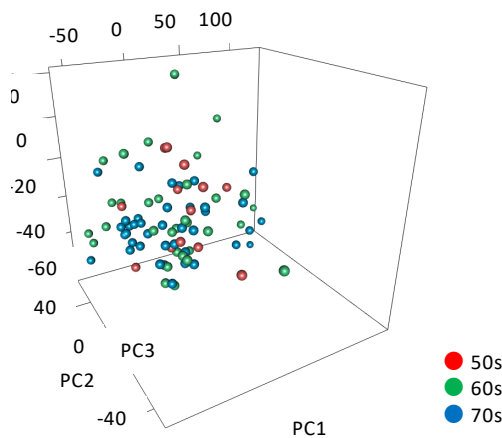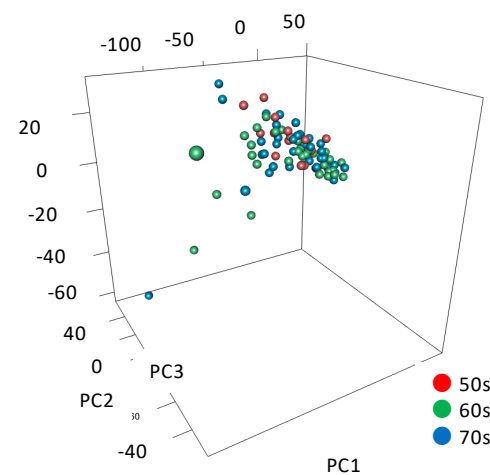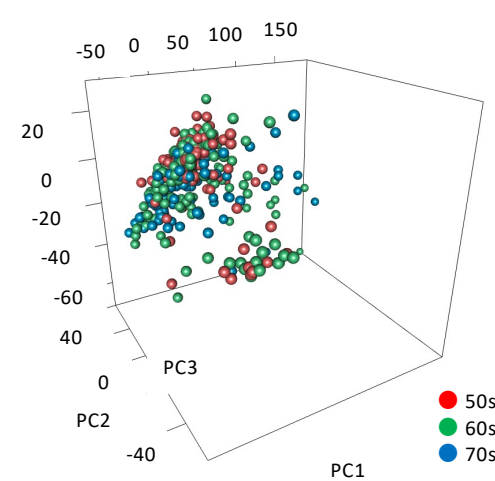

Supplemental Fig 1

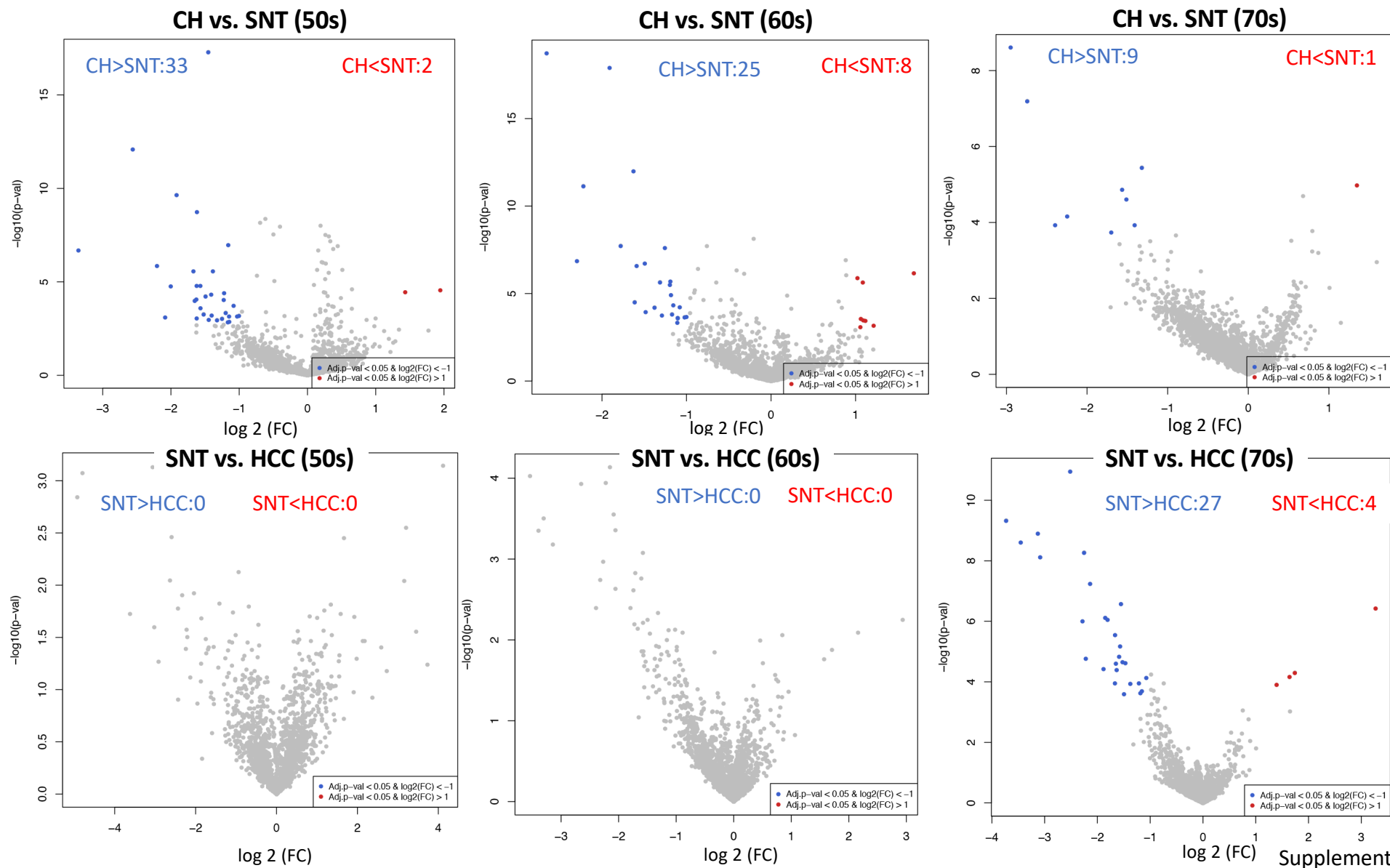

Supplemental Fig 2

Supplement: Supplementary file 1 [file ijms-25-07858-s001.zip › ijms-3061624-supplementary.pdf]
